# Stereotactic Body Radiation Therapy: A Versatile, Well-Tolerated, and Effective Treatment Option for Extracranial Metastases From Primary Ovarian and Uterine Cancer

**DOI:** 10.3389/fonc.2020.572564

**Published:** 2020-12-10

**Authors:** Nima Aghdam, Michael C. Repka, Mary McGunigal, Abby Pepin, Ima Paydar, Sonali Rudra, Nitika Paudel, Monica Pernia Marin, Simeng Suy, Sean P. Collins, Willard Barnes, Brian T. Collins

**Affiliations:** ^1^ Department of Radiation Medicine, Georgetown University Hospital, Washington, DC, United States; ^2^ Department of Radiation Oncology, Beth Israel Deaconess Medical Center and Harvard Medical School, Boston, MA, United States; ^3^ Department of Radiation Oncology, New York University Winthrop Hospital, Mineola, NY, United States; ^4^ School of Medicine and Health Sciences, George Washington University, Washington, DC, United States; ^5^ Department of Radiation Oncology, University of Pennsylvania, Philadelphia, PA, United States; ^6^ Geriatric and Palliative Medicine Division, George Washington University Hospital, Washington, DC, United States; ^7^ Division of Gynecologic Oncology, Georgetown University Hospital, Washington, DC, United States

**Keywords:** radiation, stereotactic body radiation therapy, ovarian cancer, uterine cancer, metastatic, oligometastatic

## Abstract

**Purpose:**

Single extracranial metastases from ovarian and uterine malignancies have historically been treated with surgery or conventional radiation. We report mature local control (LC), overall survival (OS), progression free survival (PFS), and toxicity for patients who completed 5-fraction stereotactic body radiation therapy (SBRT).

**Methods:**

Patients with biopsy-proven, single extracranial metastases from primary ovarian and uterine malignancies treated with 5-fraction SBRT were included. Patients were stratified based on tumor volume (small < 50 cc or large ≥ 50 cc) and dose (low dose < 35 Gy or high ≥ 35 Gy). Kaplan–Meier method was used to estimate LC, OS, and PFS.

**Results:**

Between July 2007 and July 2012, 20 patients underwent SBRT to a single extracranial metastasis. Primary site was divided evenly between ovarian and uterine (n = 10 each). Metastases involved the liver (30%), abdominal lymph nodes (25%), lung (20%), pelvic lymph nodes (10%), spine (10%), and extremity (5%). The median gross tumor volume (GTV) was 42.5 cc (range, 5–273 cc) and the median dose to the GTV was 35 Gy (range, 30–50 Gy). At a median follow-up of 56 months, the 5-year LC and OS estimates were 73 and 46%. When stratified by tumor volume, the 5-year LC and OS for small tumors were significantly better at 100% (p < 0.01) and 65% (p < 0.02). When stratified by dose, the 5-year LC was 87.5% with high dose and 53.6% with low dose (p = 0.035). The 5-year PFS for the entire cohort was 20%. Four patients with small metastases who had complete response remained disease free at study completion and were considered cured (median PFS > 10 years). Treatment was generally well tolerated, and only one patient experienced a late grade III musculoskeletal SBRT related toxicity.

**Conclusions:**

SBRT is a versatile, well-tolerated, and effective treatment option for single extracranial metastases from ovarian and uterine primary tumors. 35 Gy in five fractions appears to be a practical minimum effective dose. Four patients with small metastases were disease free at the study completion and considered cured. However, patients with larger metastases (≥50 cc) may require higher SBRT dosing or alternative treatments.

## Introduction

Ovarian and uterine cancer remain the fifth (13,980) and sixth (12,160) leading causes of cancer related death in U.S. women despite improvement in therapy (1). Systemic therapies form the cornerstone of treatment for metastatic tumors. However, aggressive local therapy in a selected patient population with an eradicated primary tumor and limited metastatic disease (≤5 metastases), has yielded promising results (2, 3). For these oligometastatic patients, metastasectomy can provide lengthy disease-free intervals in conjunction with standard systemic therapies (4).

Not all patients are surgical candidates, either due to their comorbidities or unfavorable sites of metastases. In such cases, ablative radiation therapy may prove to be an ideal local therapy. The outcomes of conventional radiation in the treatment of metastatic disease has been disappointing due to significant local failure rates, particularly for tumors with unfavorable histology such as sarcomas (5, 6). Furthermore, the delivery of the high doses necessary to eradicate gross disease in the lung, liver, and peritoneal cavity leads to unacceptable toxicity with conventional techniques.

Historically, low dose (30 Gy in 20 fractions) whole abdominal irradiation (WAI) was utilized to treat predominately radiosensitive ovarian and uterine tumors following gross total resection with good long-term local control of microscopic peritoneal disease (7–9). Adjuvant chemotherapy has now emerged as the preferred treatment for advanced ovarian and uterine malignancies following radical surgery. In the oligometastatic setting however, in order to achieve long-term disease-free survival it may be necessary to eradicate sites of chemotherapy resistant gross disease with treatments other than surgery (10). Fortunately, over the past two decades, stereotactic body radiation therapy (SBRT) has emerged as the preferred method to deliver ablative radiation doses to a wide range of extracranial tumors with exceptional precision and safety (4). Visceral metastases involving both the lung and liver have been successfully eradicated with SBRT and such treatment has prolonged survival in select groups of patients (11, 12). Further refinement to this technique has come in the form of real-time tumor tracking technologies which may enhance the safety profile by reducing the treatment margins mandatory for achieving durable local control (13, 14).

The aim of this study was to treat a selected subset of oligometastatic ovarian and uterine cancer patients with minimal disease, identify clinical features that could predict long-term disease-free survival, and establish a safe minimum effective dose in five fractions for this cohort. Here we report the mature results from 20 consecutive patients with single extracranial metastases arising from previously eradicated ovarian and uterine primary tumors treated with 5-fraction robotic SBRT.

## Materials and Methods

### Patient Selection

The Georgetown University Institutional Review Board (IRB) approved this single institution retrospective review. Consecutive patients treated per an institutional protocol who had single extracranial metastases and a controlled ovarian or uterine primary tumor were eligible for this study. Biopsy confirmation of the metastatic site and concordance with the patient’s original pathologic diagnosis were required for study inclusion. Vaginal cuff failures, multiple sites of metastases and re-irradiation were considered exclusion criteria. A single metastasis was defined as the only gross disease present following the last course of therapy. Extent and frequency of prior cytoreductive procedures or systemic agents were not exclusion criteria. Baseline PET/CT with IV contrast was performed for each patient when feasible. Eastern Cooperative Oncology Group performance status of two or less was required for inclusion. Patients were stratified based on tumor volume (small tumors < 50 cc or large tumors ≥ 50 cc) and dose (low dose < 35 Gy or high dose ≥ 35 Gy). The decision to proceed with SBRT in lieu of surgery was reached in consultation with the gynecologic oncologist (WB). All patients included in this analysis were treated with robotic SBRT in five fractions using the CyberKnife Robotic Radiosurgery System (Accuray Inc., Sunnyvale, CA, USA).

### Fiducial Placement

Tumor tracking based on translational and rotational target information routinely requires the use of a minimum of three non-colinear fiducials to be visible on the orthogonal images of the CyberKnife x-ray targeting system. Therefore, an interventional radiologist or pulmonologist inserted three to five gold fiducials *via* CT guidance or bronchoscopy in or near tumors susceptible to intrafraction motion (13). An exception to this practice is spine or paraspinal tumors, which were treated using spine tracking (14).

### Treatment Planning and Delivery

**Figure 1:**
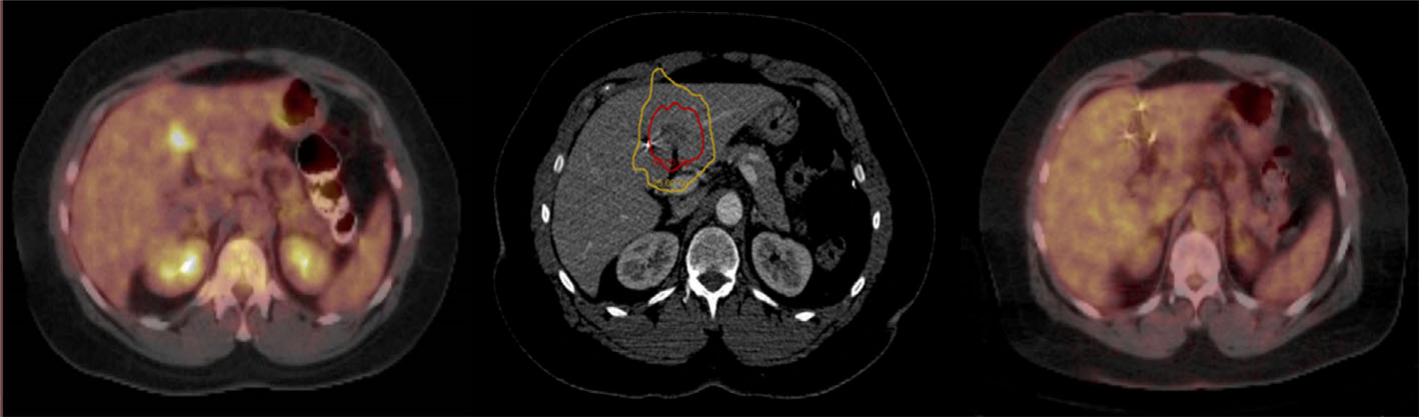
Pre-treatment PET/CT with a single liver metastasis (Left); Red prescription isodose line encompassing the liver metastasis (Center); PET/CT at 6 months post-treatment demonstrating eradication of the liver metastasis (Right).

Patients were simulated in the supine treatment position with arms at sides using high resolution (1.25 mm slices) contrast enhanced planning CT. Gross tumor volumes (GTVs) as well as organs at risk (OARs) of developing radiation damage were contoured by the treating radiation oncologist (BC). For stationary tumors, the GTV was the planning target volume (PTV). However, for lung and liver tumors that move with respiration, the GTV margin was uniformly expanded by 5 mm to establish the PTV margin. Clinical target volumes (CTVs), generally used to account for microscopic disease spread, were not used in this study. A treatment plan was generated using the MultiPlan (Accuray Inc., Sunnyvale, CA, USA) non-isocentric, inverse-planning algorithm with tissue density heterogeneity corrections for lung based on an effective depth correction (13). Treatment plans included more than 100 non-isocentric pencil beams. The radiation dose was divided into five equal fractions, prescribed to an isodose line that covered at least 95% of the PTV. Dose constraints for OARs were consistent with the widely accepted AAPM TG-101 report (15). Patients were treated per previously published detailed institutional protocols (13, 14). A representative treatment plan and tumor response are illustrated in **Figure 1**.

### Follow-Up

Patients were followed per routine institutional practice. Local failure was defined as progression of disease on CT or PET imaging as determined by the treating radiation oncologist (BC) and gynecologic oncologist (WB). Equivocal findings were dated as time of failure upon confirmation of disease progression at the next follow up imaging. Toxicities were scored according to the National Cancer Institute Common Terminology Criteria for Adverse Events, Version 4.0 (16).

### Statistical Analyses

All outcomes were calculated from the date of completion of SBRT treatment to the last date of known follow-up or death. Actuarial local control (LC), overall survival (OS), and progression free survival (PFS) were calculated and compared using the Kaplan–Meier method and log-rank test. Statistical analysis was performed using SPSS Statistics version 23.0 (IBM Corporation, Armonk, NY, USA).

## Results

### Overview

Between July 2007 and July 2012, a total of 20 patients were identified meeting study inclusion criteria. The median patient age was 64 years (range, 47–92) and the median ECOG performance status score was 0 (range, 0–1). Ninety-five percent of patients had completed chemotherapy at some point in their clinical course prior to SBRT. The median GTV was 42.5 cc (range, 5.0–273.0). The median prescription dose was 35 Gy (range, 30–50), with a median daily dose of 7 Gy (range, 6–10). All patients were treated in five fractions over a median 7-day period (range, 5–9). The median prescription isodose line was 76% (range, 70–84%), with a median maximum plan point dose of 43.7 Gy (range, 37.1–61.3 Gy). The median cumulative prescription equivalent dose in 2 Gy fractions (EQD2) was 50 Gy (*α*/*β* = 10). Patient and treatment characteristics are presented in **Table 1**.

**Table 1:** Baseline patient and treatment characteristics.

Characteristic	Median (Range)
Age (years)	64 (44–92)
ECOG PS (score)	0 (0–1)
Gross Tumor Volume (cc)	42.5 (5.0–273.0)
Prescription IDL (%)	76.0 (70.0–84.0)
Prescription Dose (Gy)	35.0 (30.0–50.0)

Primary tumor site was divided evenly between ovarian and uterine (n = 10 each). The most common histology was endometrioid adenocarcinoma (n = 6, 30%), followed by papillary serous carcinoma (n = 4, 20%). Other tumors included granulosa cell tumor (n = 2), carcinosarcoma (n = 2), clear cell carcinoma (n = 2), leiomyosarcoma (n = 2), and one mucinous adenocarcinoma and undifferentiated carcinoma each. Eleven metastases involved the abdomen, nearly evenly distributed between the liver (n = 6) and para-aortic lymph nodes (n = 5). Four metastases were located in the lung, two were located within pelvic lymph nodes, two were located within the spine and one was located in the thigh. Individual tumor characteristics are summarized in **Table 2**.

**Table 2:** Individual tumor characteristics.

Patient	Organ	Histology	Site	GTV (cc)	Dose (Gy)
1	Ovary	Granulosa Cell Tumor	Spine	10	30
2^†^	Uterus	Endometriod Adenocarcinoma	Lung	20	45
3^†^	Uterus	Endometriod Adenocarcinoma	Abdomen	5	35
4^†^	Ovary	Papillary Serous Carcinoma	Liver	20	45
5	Uterus	Endometriod Adenocarcinoma	Spine	96	35
6	Uterus	Carcinosarcoma	Abdomen	51	30
7	Uterus	Carcinosarcoma	Abdomen	30	30
8	Ovary	Clear Cell Carcinoma	Lung	46	50
9	Ovary	Undifferentiated Carcinoma	Liver	22	50
10	Ovary	Papillary Serous Carcinoma	Liver	97	35
11	Ovary	Granulosa Cell Tumor	Abdomen	39	30
12	Ovary	Mucinous Adenocarcinoma	Liver	24	35
13	Ovary	Papillary Serous Carcinoma	Liver	108	30
14	Uterus	Endometriod Adenocarcinoma	Abdomen	36	35
15	Uterus	Leiomyosarcoma	Thigh	273	40
16	Uterus	Leiomyosarcoma	Pelvis	53	30
17	Uterus	Endometriod Adenocarcinoma	Lung	63	40
18^†^	Uterus	Endometroid Adenocarcinoma	Lung	48	40
19	Ovary	Clear Cell Carcinoma	Pelvis	166	30
20	Ovary	Papillary Serous Carcinoma	Liver	28	30

^†^indicates long-term survivors.

### Local Control, Overall Survival, and Progression Free Survival 

**Figure 2:**
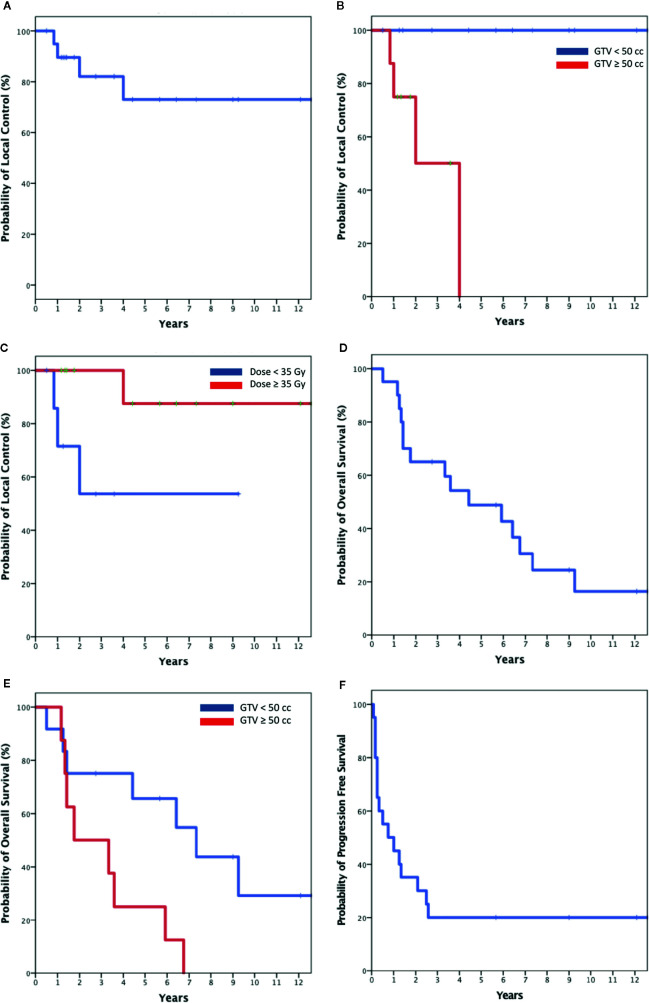
Kaplan-Meier plot of Local control for the entire cohort **(A)**; local control stratified by size **(B)** and dose **(C)**; Overall survival for the entire cohort and **(D)** and stratified by size **(E)**; progression free survival for entire cohort **(F)**.

At a median follow-up of 56 months, there were four local failures. Local control rates at 2 and 5 years were 82 and 73% (**Figure 2A**). When stratified by size, the LC at 5 years was 100% in patients with tumors < 50 cc and 0% in larger tumors (p < 0.01) (**Figure 2B**). Only one patient treated with high-dose SBRT (n = 12) experienced a local failure compared to three patients treated with low-dose SBRT (n = 8, *p* = 0.035) (**Figure 2C**). The four local failures occurred in a para-aortic lymph node, pelvic lymph node, liver, and thigh.

Median OS was 4 years. Two-year and 5-year OS where 65 and 46% (**Figure 2D**). When stratified by size OS at 5 years was 65% for smaller tumors and 25% for larger tumors (p < 0.02) (**Figure 2E**). Median PFS was 9 months. Forty-five percent of patients remained disease free at 1 year with the rates decreasing to 30 and 20% at 2 and 5 years (**Figure 2F**). All deaths were attributed to metastatic disease. At the time of the writing of this report four patients with biopsy proven metastases remain disease free and were considered cured at a median overall survival of 126 months (range, 68–151 months) following SBRT alone. These tumors were relatively small (median size 20 cc), involved favorable locations including the lung (2), liver and paraaortic lymph node and received a relatively high mean dose of 40 Gy (range, 35–45). Three of the cured tumors where relatively indolent endometrioid adenocarcinoma metastases, and one was a potentially more aggressive liver ovarian papillary serous carcinoma metastasis in a BRCA mutation free patient (**Table 2**).

### Toxicity

Overall, the treatment was well tolerated with no acute grade III or higher toxicity and only one reported late grade III toxicity. The most common acute toxicities were fatigue (n = 5) and nausea (n = 5). Nausea requiring medication occurred in three patients with abdominal lesions (n = 3). One late lumbar vertebral body compression fracture was observed approximately 7 years following the treatment of a para-aortic lymph node metastasis (30 Gy) in a patient with osteopenia.

## Discussion

In the earliest gynecological oligometastatic SBRT prospective trial of its kind, Kunos et al. treated 50 patients with a wide variety of primary tumors between July 2009 and September 2011 (17). The majority were ovarian adenocarcinoma (50%) or uterine adenocarcionoma (28%) with the remainder being either squamous cell carcinoma of the cervix (18%) or vulva (4%). Only 28% of the patients had single metastases. Most patients had two or three metastases (68%) involving lymph nodes (68%). However, metastases involving the liver (16%), lung (8%), and spine (4%) were also treated. Prior chemotherapy (94%), adjuvant chemotherapy (8%), and re-irradiation (32%) were permitted. Appropriately, for such a diverse group of patients and targets being treated on a Phase II clinical trial, a relatively low dose of 24 Gy in three fractions (prescription EQD2 = 36 Gy with *α*/*β*=10) was safely delivered. Surprisingly, no SBRT targeted disease progressed during the fairly brief 15-month median follow-up period for surviving patients (range, 1–31 months). Nonetheless, median PFS and OS were only 7.8 and 20.2 months prompting the investigators to explore combined modality approaches for this patient population.

Recently, Lazzari et al. reported their oligometastatic ovarian cancer SBRT outcomes (18). Eighty two patients with 156 metastases were treated between May 2012 and December 2016. A sizable majority of patients had single metastases (70.7%) involving lymph nodes (72%). However, metastases involving the liver (9%) and lung (4%) were also treated. Metastases were generally small with a median GTV of 6.77 cc (range, 0.19–90.50 cc). Prior chemotherapy (98.8%), concomitant chemotherapy (18.6%), and re-irradiation (3.2%) were permitted. A relatively low median prescription dose of 24 Gy (range, 14–45 Gy) was generally delivered in three fractions (range, 1–5 fractions) with limited acute toxicity. At a median follow-up of 17.4 months, the actuarial 2-year local progression-free survival was 68%. However, OS at 2 years was 71%, likely resulting from the high number of patients with small single lymph node metastases. Nonetheless, the median systemic treatment-free interval after SBRT was 7.4 months, mirroring the Kunos et al. experience.

Our series differs from contemporaneous SBRT series in that all patients were required to have a single metastasis, and re-irradiation was not permited (17, 18). The single metastasis requirement was necessary in order to lengthen our cohorts’ OS allowing for the testing of our proposed hypotheses. The exclusion of re-irradiation patients was necessary so as not to interfere with our planned minimum effective SBRT dose analysis. Consistent with contemporaneous SBRT studies, the majority of our patients completed chemotherapy prior to SBRT (95%). Over a 5-year period extending from July 2007 to July 2012, we treated 20 patients with single biopsy proven ovarian (50%) and uterine (50%) cancer oligometastases. Half of the metastases involved mobile visceral organs including the liver (30%) and lung (20%). The other half involved static lymph nodes (35%) or musculoskeletal structures (15%). At a median follow-up of 56 months, our 2-year and 5-year OS were 65 and 46%, justifying our selection criteria.

First, we hypothesized that a potentially curable oligometastatic state exists in patients with single extracranial ovarian and uterine cancer metastases and that these patients are potentially curable with SBRT alone (19). At a median follow-up of 56 months (range, 6–151 months), the 5-year LC and OS estimates were a reasonable 73 and 46%. However, when stratified by GTV, the 5-year LC and OS for small tumors (<50 cc) were significantly better at 100% (p < 0.01) and 65% (p < 0.02). Furthermore, at the time of the writing of this report, four biopsy proven oligometastatic patients (20% of the study cohort) remain alive and disease free at a median follow-up of greater than 10 years without post-treatment systemic therapy further bolstering our hypothesis. These four patient’s metastases were relatively small with a median volume of 20 cc (range, 5–48 cc), of fairly indolent histology (75% endometroid adenocarcinoma/25% papillary serous carcinoma) and involved favorable locations for high-dose SBRT delivery (median dose 40 Gy) as illustrated in **Table 2**. Our research suggests that future studies should at a minimum evaluate the role of SBRT in the treatment of small endometroid adenocarcinoma and ovarian papillary serous carcinoma oligometastases. It appears that at least a small minority of these patients are curable with SBRT alone.

Second, we hypothesized that a safe broadly applicable minimal effective SBRT dose in five fractions exists for extracranial ovarian and uterine oligometastases. Our median prescription dose was 35 Gy (range, 30–50) and our local control rates at 2 and 5 years were 82 and 73%. Three of the eight patients who were treated with 30 Gy in five fractions in our series failed locally; only one of 12 patients in the high-dose arm (≥35 Gy) experienced local failure. Our current practice is to treat patients with a minimum dose of 35 Gy in five fractions. In our experience normal tissue dose constraints can be routinely met with this approach limiting acute and chronic toxicity. However, when safe, we dose escalate in all patients respecting published dose constraints (15). We consider dose escalation particularly important for larger tumors (≥50 cc) that are not resectable.

Finally, treating single ovarian and uterine oligometastases did not meaningfully increase our PFS relative to contemporaneous studies. Median PFS was just 9 months. As previously advocated by Kunos et al., in order to improve cure rates, identifying effective systemic agents to pair with SBRT is of paramount importance (17). With the recent proliferation of novel immuno and molecular therapies there are now a large number of drugs worthy of study. Nonetheless, in patients who have been extensively treated with systemic agents and would likely continue to receive treatment for the remainder of their lives, we agree with Lazzari et al. that even a brief chemotherapy free interval may be a valuable endpoint in and of itself (18).

Limitations of this series include the fact that this is a single-institution, retrospective study with a relatively small patient population. Although one of the aims was to identify clinical factors that could significantly predict PFS, the limited sample size precluded this. Additionally, the relative rarity of the single metastasis presentation, diversity of primary and metastatic disease sites treated and pre- and post-SBRT systemic therapy regimens used confounds the interpretation of our outcome data. On the first point, we wished to test the hypothesis that SBRT may cure patients in the earliest stages of metastatic disease. Our long follow-up and suitable OS supports this selection criteria. However, it is possible that the presence of a single metastasis suggests a relatively indolent disease entity relative to patients with two or more metastases. Our median OS being a lengthy 4 years while our median PFS is only a mere 9 months supports this valid criticism. We concede that our results may only be applicable to patients with single metastases and not the more commonly observed oligometastatic patient with two or three metastases. Also, the diversity of tumors irradiated with varied SBRT protocols and a myriad of systemic agents used before and after treatment make it difficult to evaluate the local efficacy and clinical impact of SBRT on this patient population. Future trials, if feasible, should treat well defined not previously treated disease entities such as oligometastatic endometroid adenocarcinoma with standard SBRT and systemic therapy protocols.

## Conclusion

The 5-year OS (46%) and PFS (20%) reported in this manuscript closely resemble the recently reported SABR-COMET trials significantly improved 5-year OS (42.3%) and PFS (17.3%) for predominately breast, lung, colorectal, and prostate oligometastatic patients treated with SBRT (20). Our results suggest that SBRT is also a safe and effective treatment for oligometastatic ovarian and uterine cancer patients. Furthermore, the long-term disease-free survivors in our cohort support the presence of a true oligometastatic state in ovarian and uterine cancer curable with SBRT alone (19). Durable local control is achievable in most metastases with a minimum dose of 35 Gy. A safe, widely applicable five fraction scheme minimizes needless toxicity in a group of patients likely to develop additional metastatic sites and ultimately die from metastatic disease. Finally, a reasonable disease-free interval (median 9 months) after such a brief local therapy (median 7 days) allows patients to quickly pursue new systemic agents if deemed necessary or recover from prior treatment (18). Further refinement of patient selection and SBRT protocols as well as the identification of suitable concurrent agents will be required to improve outcomes in the future (17).

## Data Availability Statement

The datasets presented in this article are not readily available because: The authors are unable to share the data as we did not include data sharing provisions in the IRB (2011-210). Requests to access the datasets should be directed to collinsb@gunet.georgetown.edu.

## Ethics Statement

This retrospective study was approved by Georgetown University institutional review board (IRB-2011-210).

## Author Contributions

NA and BC contributed to conception and design of the study. BC and WB contributed to data collection. NA performed data statistical analysis. All authors contributed to the article and approved the submitted version.

## Conflict of Interest

SC and BC serve as clinical consultants to Accuray Inc. The Department of Radiation Medicine at Georgetown University Hospital receives a grant from Accuray to support a research coordinator.

The remaining authors declare that the research was conducted in the absence of any commercial or financial relationships that could be construed as a potential conflict of interest.
